# The Use of “Omics” in Lactation Research in Dairy Cows

**DOI:** 10.3390/ijms18050983

**Published:** 2017-05-05

**Authors:** Shanshan Li, Quanjuan Wang, Xiujuan Lin, Xiaolu Jin, Lan Liu, Caihong Wang, Qiong Chen, Jianxin Liu, Hongyun Liu

**Affiliations:** Institute of Dairy Science, College of Animal Sciences, Zhejiang University, Hangzhou 310058, China; leeshanshanshan@163.com (S.L.);18867116391@sina.cn (Q.W.); ambrose123456@163.com (X.L.); chnfhjxl@163.com (X.J.); liulan11279@gmail.com (L.L.); 11617003@zju.edu.cn (C.W.); qchen1207@163.com (Q.C.); liujx@zju.edu.cn (J.L.)

**Keywords:** genomics, transcriptomics, proteomics, metabolomics, lactation

## Abstract

“Omics” is the application of genomics, transcriptomics, proteomics, and metabolomics in biological research. Over the years, tremendous amounts of biological information has been gathered regarding the changes in gene, mRNA and protein expressions as well as metabolites in different physiological conditions and regulations, which has greatly advanced our understanding of the regulation of many physiological and pathophysiological processes. The aim of this review is to comprehensively describe the advances in our knowledge regarding lactation mainly in dairy cows that were obtained from the “omics” studies. The “omics” technologies have continuously been preferred as the technical tools in lactation research aiming to develop new nutritional, genetic, and management strategies to improve milk production and milk quality in dairy cows.

## 1. Introduction

Lactation has been widely investigated for years at the morphological, physiological and even genetic levels due to its important nutritional functions [1,2,3,4,5]. Lactation is also a complex biological activity; its initiation and maintenance involve general biochemical and endocrinological processes in the mammary glands [6]. To improve milk quality and milk production performance, major advances in understanding the physiology of lactating mammary glands, such as technologies and theories, have taken place in the past few decades.

**Figure 1:**
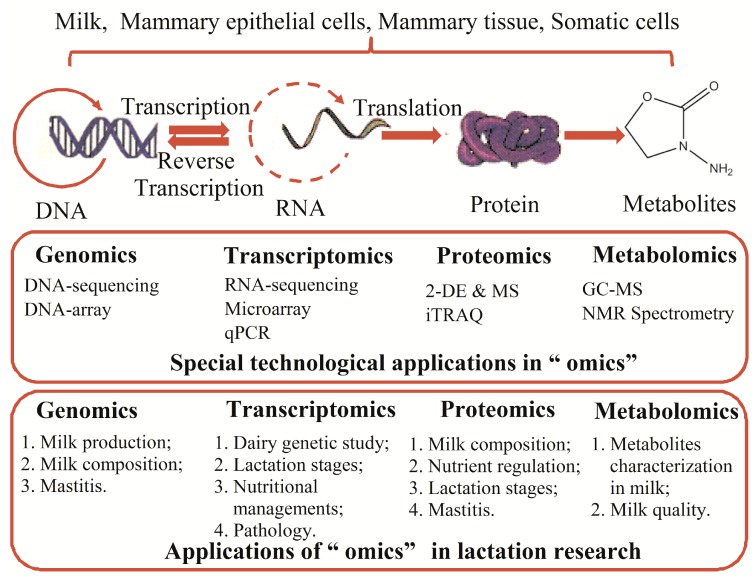
The methods applied in omics research and their applications in lactation research. Abbreviations: 2-DE, two-dimensional electrophoresis; MS, mass spectrometry; iTRAQ, isobaric tags for absolute and relative quantification; GC-MS, gas chromatography-mass spectrometry; NMR, nuclear magnetic resonance. The arrows refer to the reactive direction.

Previous studies have introduced the changes in the alveoli, ducts and stroma during the development of the mammary gland, which may help guide us in developing a perfect plan to manage or feed the lactating cows [3]. A great number of genes are differentially expressed between the non-lactation and lactation states, and these expression alterations may play crucial roles in the regulation of lactation [7]. Therefore, recently developed “omics” technologies make it possible to comprehensively and systematically study lactation at the levels of DNA, RNA, proteins, and metabolites and to identify the most important factors or processes that may influence lactation. Genomics not only maps the genes but also performs sequence analysis to provide insights into the coding gene heterogeneity [8]. Transcriptomics can simultaneously profile several to more than thousands of mRNA expression levels and changes [8]. The proteomic tools are used to determine the changes in protein expression patterns, abundance, and post-translational modifications [9]. Moreover, metabolomics technology has been used to analyze large groups of metabolites in biological samples that can reflect the physicochemical properties of the body [10]. By applying these new technologies, we have stepped forward tremendously in our knowledge and understanding of lactation physiology and pathology, which is described below and summarized in Figure 1.

## 2. Genomics in Lactation Research

Genomics has been regarded as an effective method to study lactation; it involves genome mapping of all the genes (i.e., genetic maps, physical maps, and transcript maps), gene sequence analysis, and gene functional analysis. Genomics helps us select breeds for ideal traits, develop new nutritional strategies for better production performance, and even effectively predict mammary diseases. Unlike the previous publication by Seo et al. [11], which mainly discussed the potential nutritional strategies for dairy cattle based on genomic studies, here we summarized the previous applications of genomics in lactation research (see Table 1 for details), especially in dairy cows.

### 2.1. Special Technological Applications in Genomic Studies

There are two common methods of inferring genomics: DNA sequencing and high-density microarray analysis. The classic DNA sequencing methods, such as Sanger sequencing, are accurate, but time-consuming and low-throughput [20]. Some animals, such as dairy cows, were fully sequenced by this method due to their significant economic value [21]. Because of the rapid development of bioinformatics, computation and instrumentation, next-generation DNA sequencing has become the essential technology to explore genetic problems, and it significantly drives the progress of biology [22]. Shendure et al. [20] and Reis-Filho [22] showed that the new next sequencing technology has overcome above weaknesses of classic DNA sequencing technology and become inexpensive, common and widespread. In addition, another method is DNA microarray, which is mainly applied to identify single nucleotide polymorphisms (SNPs) or mutations and RNA abundance [8]. These two technologies have specific properties that should be taken into consideration and both have been applied widely in dairy cows lactation research.

### 2.2. Applications of Genomics in Lactation Research

#### 2.2.1. Genomic Studies in Milk Production

Milk production is an important aspect of lactation, which is usually affected by factors such as environment, nutritional management, breed, etc. [23]. The changes in gene expression have great significance in milk production. Many studies have focused on detecting the relationships between milk production traits and the candidate genes associated with milk protein and fat yields as well as their percentages [24,25]. It has been commonly shown that most production traits are varied in the persistent period after the peak, which is influenced by genes [26]. The ras-related protein rap-1A on bovine chromosome 3, and insulin-like growth factor 2 are found to be strong candidate genes for the quantitative trait locus (QTL), which may affect milk production [12,13]. In addition, researchers have combined the genetic data with dry matter intake, live weight, fat-protein-corrected milk yield and body condition score during different lactation periods, which may contribute to the efficient prediction of traits in lactation [27,28].

However, the available data sets of genomics research in milk production were relatively scarce in the past few years, which has made it hard and costly to measure milk protein and fat yields as well as their respective percentages, urea nitrogen yield, etc. Fortunately, based on pedigree or/and SNP, the relationships between animals made it possible to estimate the genetic parameters [29]. Veerkamp et al. developed a parametric bootstrapping procedure using the small data to accurately estimate the heritability and genetic correlations between traits through genomic and pedigree relationships instead of phenotypes [29]. Moreover, genome-wide analysis has been used to detect biomarkers associated with reproduction and has gradually become a better way to explain key genes and pathways. For example, the gene polymorphisms of epidermal growth factor (EGF) and signal transducer and activator of transcription 5A (STAT5A), a missense mutation of prophet of Pit 1 (PROP1), and the deletion of Ribonuclease H2 Subunit B (RNASEH2B) were associated with milk production [30]. Overall, genomic selection will be a promising approach to estimating breeding values in the future.

#### 2.2.2. Genomic Studies in Milk Composition

Milk fat and protein are the major nutritional components associated with milk quality. Genomic studies have found links between specific genes and milk fat composition. An association analysis of SNPs discovered that 54 regions on the 29 chromosomes are significantly linked to at least one fatty acid in Dutch dairy cattle [31]. Diacylglycerol-acyl transferase 1 (DGAT1) is linked to multiple milk traits and is highly correlated with fat yield and fat percentage [14,15]. In addition, the DGAT1 K232A and stearoyl CoA desaturase 1 A293 V polymorphisms are associated with milk fat composition in both winter and summer [16]. Additionally, Bauman et al. [32] studied diet-induced milk fat depression and indicated that fatty acids such as trans-10 and cis-12 conjugated linoleic acidare associated with lipid synthesis and can directly reduce sterol response element-binding protein-1, a transcriptional factor that plays a central role in responding to the lipogenic signaling pathway. These studies are conducive to regulating milk fat composition through breeding and genetic manipulations based on the genetic superiority of milk fat composition.

Genomic studies have also found specific genes linked to milk protein composition, which is affected by several factors, such as the season, lactation stage, diet and disease, but especially genes [33]. Therefore, the understanding of genes that code milk protein should be advanced, and such information will contribute to breed selection. For example, from an estimated 1912 Dutch Holstein Friesian cows, the β-casein (CN) genotype and the κ-CN genotype have major impacts on protein yield and percentage, respectively. Both variants of the β-lactoglobulin (LG) genotype B and the β-κ-CN haplotype A2B are fit for cheese production [17,18]. These results greatly enable us to assess the correlation of breeding to milk protein composition and better design the breeding plans before we implement them.

#### 2.2.3. Genomic Studies in Mastitis

Mastitis enormously influences the dairy industry and causes significant economic losses because of the decline of milk production, veterinary costs, and discarded milk [34]. High somatic cell count (SCC) is a hallmark of mastitis. Recently, genomics has been widely exploited to look into mastitis. For instance, SNPs on Bos Taurus autosome 4 (BTA4) and BTA18 are found to be significantly associated with lactation-average SCC, while an SNP on BTA6 is associated with the standard deviation of test-day SCC score [19]. The GeneMania gene network analysis also discovered a co-expression network containing 665 interactions in the 145 genes and recognized a candidate QTL for clinical mastitis in the US Holstein population [35]. These observations can help to identify the genetic mechanisms leading to mastitis and new targets for mastitis therapeutics.

## 3. Transcriptomics in Lactation Research

Transcriptomics, a continuously growing field in the studies of lactation, can be classified based on the RNA types (mRNA and non-coding RNA) or methodology (microarray and RNA sequencing technology). There are numerous studies that are focused on mRNA expression profiles to look for functional genes during lactation, especially in bovine mammary glands. For example, mRNA differential expression revealed candidate genes for breeding [36]. The miRNAs and long non-coding RNAs are also required for mammary gland development and lactation [37,38,39]. Furthermore, transcriptomic studies have been used to investigate the mechanisms of mammary gland development, effects of nutritional management on milk synthesis, mammary transcriptional response to pathology, etc. [40,41,42].

### 3.1. Special Technological Applications in Transcriptomics

#### 3.1.1. Methods of Transcriptomics in Lactation Research

Similar to genomics, there are mainly two study methods of transcriptomics: microarray and RNA sequencing (RNA-seq). With the advancement of technology, RNA-seq has overcome the weaknesses of Sanger sequencing, became increasingly universal, and revolutionized advances in eukaryotic transcriptomic analysis [43]. Researchers obtain total sequence reads (35–500 bp) from next-generation sequencing platforms such as Illumina, SOLiD and 454 and then reconstruct the transcripts by transcriptome assembly [44]. Both of these transcriptomics methods have been widely utilized in lactation research [45,46], although the RNA-seq method may replace microarray, as it can reveal the complex landscapes and transcriptional dynamics from yeast to humans and show high sensitivity and accuracy [44].

#### 3.1.2. RNA Preparation in Lactation Transcriptomics

RNA preparation significantly influences the analysis of the transcriptome because the quality and quantity of RNA determine the reliability and reproducibility of findings. In transcriptional investigations, total RNA is mainly extracted from the mammary tissue or milk epithelial cells (see Table 2 for more details). Cánovas et al. [47] compared the representation of genes and their expression levels from all of the above sources and recommended that milk somatic cells and milk fat globules transcriptomes are an effective and easy ways to study lactation. Laser-capture micro dissection has been used to selectively isolate epithelial cells from mammary gland frozen tissue sections [48], which will greatly contribute to the future research in lactation.

### 3.2. Applications of Transcriptomics in Lactation Research

#### 3.2.1. Transcriptomics in Dairy Genetic Study

Despite some advantages in DNA-based, marker-assisted selection for traditional dairy cattle breeding programs, transcriptomic profiles still offer new opportunities to obtain functional candidate genes for specific lactation traits in dairy cows [36]. By transcriptomics, SNPs (≥33,000) associating with lactation have been discovered, which will develop the platforms of genotyping to study marker-trait associations in dairy cows [45]. In addition, there are a total of 31 differentially expressed genes between extremely high and low milk protein and fat percentages in Holstein cows. These genes are highly correlated with specific biologic processes such as mammary gland development as well as fat and protein metabolism [36]. Moreover, integrated QTL and genome wide association studies have indicated that parathyroid hormone-like hormone, ribosomal protein L23a, serum amyloid A (SAA1, Mammary-SAA3.2 and SAA3), tribbles homolog 3 and vascular endothelial growth factor A are potential genes that may influence the protein and fat percentage in milk [36]. In this context, transcriptomics is regarded as a tool to analyze the specific candidate genes associated with lactation traits and will strongly affect the breeding selection and improvement of dairy cows.

#### 3.2.2. Transcriptomics in Lactation Stages

Integrated functional studies of the transcriptome have been carried out in Holstein dairy cows to detect the cellular adaptations required for the synthesis and secretion of milk [52]. During the lactation cycle, the mammary gland usually undergoes epithelial cell proliferation and differentiation, milk synthesis, involution, and remodeling corresponding to pregnancy, the lactation period and the dry period. Alterations of the transcriptomic characteristics in mammary gland represent the adaptations of the expression profile correlated to the lactation stages (Table 3), therefore giving insights into the lactation mechanism and nutritional managements in dairy cows. The analysis of expression profiles showed that bovine mammary glands mainly concentrate on milk synthesis and cell proliferation inhibition during the onset of lactation [40]. During the whole lactation cycle, pivotal roles for insulin induced gene 1, peroxisome proliferator-activated receptor γ (PPAR) and PPAR γ coactivator 1 α in regulating lipid synthesis and insulin as well as being a mechanistic target of rapamycin for milk protein synthesis have been found [4,5]. In addition, Li et al. [7] found that 884 unique sequences of miRNA in bovine mammary gland and 56 differentially expressed miRNAs between non-lactating and lactating mammary glands, which also suggested that the expression level and types of miRNAs differ between these two periods in dairy cows. The network analysis described the interactions of the reported miRNAs associated with lactation and their target genes, thus furthering the understanding of the function of these miRNAs [7]. The differentially gene expression also was investigated using RNA-seq-based transcriptomic analysis during non-lactating and lactation in dairy cows. The increased gene expression in lactating mammary gland included genes related with various macromolecular metabolic processes, and appeared to promote greater metabolic activity associated with milk synthesis and secretion [53]. Such analyses offer us key information and greatly contribute to the study of miRNA and gene expression in lactation. However, more transcriptomic studies should be done on dairy cows, as were performed on mice’s mammary gland [54].

#### 3.2.3. Transcriptomics in Lactation Relating to Nutrition and Management

Transcriptomics can be used to explore the underlying mechanisms of many factors, including diets, milking frequency, and photoperiod, on lactation. For example, short-term restricted feeding or food-deprivation differentially regulates milk secretion and mammary gene expression [41,62]. A total of 554 transcripts (423 upregulated and 131 downregulated) and 1631 transcripts (1046 upregulated and 585 downregulated) were found to be differentially expressed in mammary gland tissues when fed on low-quality (rice straw, RS [63]; or corn stover, CS [64]) and high-quality forages (alfalfa hay, AH). Moreover, experiments have shown that the low-quality-forage-induced milk protein reduction in dairy cows might be associated with the decreased ability of protein synthesis, increased degradation of protein, and inefficiency of energy utilization, which may help us better understanding of the regulatory mechanisms underlying milk production under low-quality forage. For sucking frequency, the decrease in sucking frequency down-regulates milk synthesis and induces the pathway of apoptosis and the reconstruction of udder tissue in dairy cows [46], while increasing milking frequency contributes to more milk production [65]. Moreover, increased milking frequency changes the expression of genes involved in nutrient transportation, metabolism, proliferation and differentiation, reconstruction of extracellular matrix, κ-CN, α-lactoalbumin, etc. [66,67]. However, reduced milk-removal frequency changes gene expression associated with apoptosis, mechanical stress and epithelial tight junction synthesis [46,68]. In addition, researchers have studied the differences in performance induced by different photoperiods during late gestation and found a rise in mammary development and immune function in dairy cows that were treated with short day photoperiods [69]. Therefore, the transcriptomics may help us to understand the effects and mechanisms of feeding and management on lactation in dairy cows.

#### 3.2.4. Mammary Transcriptional Response to Pathology

Several researchers have used transcriptomics methods to study gene expression changes in the tissues (mammary gland and the periphery) and some immunocytes (macrophage and neutrophils) responsive to pathogens caused by mastitis, and these studies of bovine mammary glands have been well reviewed by Loor et al. [42].

## 4. Proteomics in Lactation Research

Proteomics is the large-scale study of proteins and is commonly defined as protein expression from gene transcription and translation. This technology can be used to characterize the diversity of the protein structure and the relationship between this diversity and underlying biological processes, which is even applied to potential disease and nutrition studies [9,70]. In fact, proteomics is a more direct way to study biological processes than genomics and transcriptomics. Proteomics has been widely used to determine the milk components, protein profiles and characteristics of milk, nutrients and lactation stages on milk synthesis, and mammary proteomic responses to mastitis in dairy cows (Table 4) [71,72,73,74,75].

### 4.1. Special Technological Applications in Proteomics

Generally, three methods are commonly used in the research of proteomics: the specific digestion of proteins [9], direct analysis of proteins after chromatographic separation [9] and mass spectrometry (MS) based quantitative technology [90]. Traditionally, the proteome was first analyzed by two-dimensional electrophoresis (2-DE) followed by MS technology. The 2-DE method has gradually been considered an “ancient” technique due to its low reproducibility and reliability of protein separation [9]. Previously, this classic proteome method was usually applied to identify the biomarkers related to diet or treatments [8,91]. Currently, quantitative proteomics, such as stable isotope labeling with amino acids in cell culture (SILAC) and isobaric tags for absolute and relative quantification (iTRAQ) combined with MS, is challenging the traditional 2-DE and MS methods because it allows for the massive multiplexing of primary data with better quality than established methods, for example, determining the protein constituents of a biological system [90,92]. The iTRAQ can be commonly employed to analyze the multiplicity of different biological samples, however, the accuracy might be compromised due to the influence of near isobaric ions contamination in a sample [90,92]. The great advantages of SILAC are simple, accurate and reproductive, which contributes to studying the characterization of proteomic profile from various biological samples, the interactions of protein-protein, the dynamic changes after protein posttranslational modification, and protein turnover at the proteome-wide level [90,92,93,94]. In addition, 2-DE combined with MS and iTRAQ have been widely applied in lactation research, particularly in the studies of mastitis, biomarkers and effects of nutrition on lactation [87,95]. Shotgun proteomics through iTRAQ makes it possible to get a better view of mammary development and function [76,77].

### 4.2. Applications of Proteomics in Lactation Research

#### 4.2.1. The Profile and Characteristics of Milk Components

In recent decades, proteomics has served as an attractive research approach to study milk protein expression in dairy cows, i.e., the milk fat globule membrane (MFGM) components and milk protein profile [76,79,96]. Reinhardt and Lippolis [76] studied MFGM in mid-lactation and identified 120 proteins that are mainly associated with cell signaling and membrane/protein trafficking. With shotgun proteomics, Reinhardt and Lippolis [77] found that the proteins associated with lipid transportation, synthesis and secretion are up-regulated, whereas apolipoproteins A1, A-IV, C-III, and E are down-regulated in day 7 MFGM after calving compared with colostrum. Moreover, during the period of early lactation, the whey proteome alters. In particular, at 48 h postpartum, both immunoglobulins and caseins are significantly diminished, while lower molecular mass proteins are increased [78]. Interestingly, the four whey proteins (β2-microglobulin, Vitamin D-binding protein, zinc-α-2-glycoprotein and immunoglobulin G2 chain C) were only detected in the colostrum, indicating that these whey proteins play critical roles in the protection of newborns [78,80].

Major differences between the same milk proteins among different species have also been shown in proteomics studies. Hinz et al. [79] found that the main whey protein in the milk from caprine, bovine, equine and buffalo is β-LG, which is only deprived in camel milk. The proteome comparisons among species may help to find adulterated milk products or samples and identify the sources of hypoallergenic replacements of bovine milk [79]. With the iTRAQ, specific proteins are regarded as characterization traits for specific species, such as clusterin (buffalo), biglycan (goat), quinone oxidoreductase and whey acidic protein (camel), clusterin (buffalo), primary amine oxidase (cow), uncharacterized protein (yak) [81], high abundance of antimicrobial proteins (bovine), and high concentrations of the mucosal defense system (human) [82]. Moreover, the study of the MFCM in different species has been performed to develop a comprehensive overview as well as distinctive profiling of the MFGM proteome among species [97]. These differences between species may be related to differences in heredity, the development of the immune system and the diet received by animals.

#### 4.2.2. Proteomics in Lactation with Different Nutrition and at Different Lactation Stages

Proteomics technology has revealed that the compositions and artifactitious pattern of the diet and restricted or elevated feeding resulted in changes in milk composition, which suggests that these factors influence the synthesis and secretion of milk in mammary glands [72,95,98]. Compared with AH forage, rice straw (RS) or corn stover (CS) forage induced milk production by affecting the expression of proteins in mammary gland tissues, i.e., 231 up-regulated and 286 down-regulated proteins (RS forage) [63], 138 up-regulated and 208 down-regulated proteins (CS forage) [64]. Using proteomics, Lu et al. identified phosphoproteins involved in the synthesis of milk proteins in the mammary epithelial cells treated with Lys or Met, which are related to a series of biological processes such as transcription, translation, protein synthesis, cell division and differentiation and even the cell cycle [83,84].

2-DE and MS were employed to compare the protein profiles between the lactation and non-lactation stages. The results concluded that the 57 up-regulated proteins are involved in biological processes such as transportation, metabolism, biosynthesis, protein processing, the pentose-phosphate shunt, secretion and cell apoptosis, while three down-regulated proteins play roles in other processes such as lipid degradation, transportation and the cytoskeleton. These proteins may play roles in the initiation, maintenance and involution of lactation [85]. Moreover, proteomic methods can identify the biomarkers for physiological imbalance (PI). Isocitrate dehydrogenase and pyruvate carboxylase were found to be the candidate biomarkers for early lactation, and during mid-lactation, while methylmalonate-semialdehyde dehydrogenase and alcohol dehydrogenase-4 were biomarkers in liver for PI [86]. The galactose-1-phosphate and stomatin in the milk are indicators for energy balance [73]. All of these investigations indicated that protein profiles and the characteristics of milk and mammary glands are excellent indicators of nutritional status and lactation stage.

#### 4.2.3. Biomarkers for Mastitis

The challenges in the identification and quantification of biomarkers make its discovery difficult [74]. To better understand mastitis, proteomics data have been used to find unique markers. Low-abundance proteins such as lactoferrin, transferrin, apolipoprotein AI, and fibrinogen were identified to be associated with inflammation [74]. After infection with *Staphylococcus aureus*, casein peptides, osteopontin, serum proteins, minor acute phase proteins and complement components were altered in the milk whey proteins [87]. In addition, the heat shock protein family and the protocadherin γ family varied in milk exosomes [87]. In addition, the neutrophil extracellular traps proteome/antimicrobial peptides have a potent antimicrobial function [87]. Research also showed that N-linked glycosylated proteins between the healthy and E. coli mastitis groups were related to immunity, stress and cell adhesion [75]. Proteins related to inflammation, such as hemoglobin β, cytochrome C oxidase, annexin V and α-1-acid glycoprotein as well as collagen type I α 1 and inter-α (Globulin) inhibitor H4, were detected to be more abundant in cows with clinical mastitis [23,88,89]. These up-regulated proteins may be associated with the damage and repair of tissue, which might be candidate genes, indicators or biomarkers for the sensitivity to mastitis [89].

## 5. Metabolomics in Lactation Research

Metabolomics is the study that adopts biochemical analytical techniques to characterize the changes of small molecular metabolites (<1500 Da) to reveal the animals’ physiological states [10]. Even though metabolomics lags behind the other omics, it attracts more and more scientists into the field because of its advantages and usefulness [99].

### 5.1. Special Technological Applications in Metabolomics

Many technologies such as MS instruments, nuclear magnetic resonance (NMR) spectrometers, capillary electrophoresis and ultra-high-pressure liquid chromatography systems are useful tools for metabolite identification [100]. In particular, MS and NMR are the most commonly used methods [100,101,102]. MS has the benefit of relatively higher sensitivity compared to NMR technology, while NMR makes it possible to analyze the samples directly and reliably with little sample preparation [103]. Wishart et al. [100] have compared the advantages and disadvantages of three analytical technologies, NMR, gas chromatography (GC)-MS, and liquid chromatography (LC)-MS. They reported that NMR requires a shorter detection time of 2–3 min per sample and has the ability to identify novel chemicals, while GC-MS and LC-MS need 20–30 min to detect each sample. Relative to NMR and GC-MS, LC-MS has superb sensitivity but less robust and mature instrumentation. However, GC-MS is relatively inexpensive compared to NMR and LC-MS [100]. Therefore, these economic or technical characteristics should be taken into consideration when researchers choose the method to study metabolites.

### 5.2. Applications of Metabolomics in Lactation Research

#### 5.2.1. Metabolite Characterization in Milk

Small molecular metabolites in milk can reflect milk quality, feeding conditions and the metabolism of cows. Our previous research proved that metabolomics could aid in the discovery of the biomarkers of milk production or milk quality, thus providing the possibility of discovering new biomarkers in dairy cows [104]. Recently, NMR or MS analysis demonstrated that biomarkers (i.e., high levels of phosphorylated saccharides, acetone and β-hydroxybutyrate) are closely correlated with the metabolic status in dairy cows during early lactation, which may help in selecting cows that cope well with the metabolic stress in early lactation [103,105].

Boudonck et al. [106] performed an interesting study to characterize the biochemical alterations of milk and found that there are higher lipid metabolites (free fatty acids, cholesterol and 1,2-dipalmitoylglycerol) and vitamin E in whole milk compared to reduced fat and fat-free milk. Hippurate and ribose 5-phosphate can be considered as markers to distinguish organic whole milk from conventional milk. Moreover, metabolites, such as choline, *N*-acetyl hexosamines, creatinine, glycerophosphocholine, glutamate, glucose 1-phosphate, galactose 1-phosphate and orotate, were identified to correlate with total protein content, while levels of lactate, acetate, glutamate, creatinine, choline, carnitine, galactose 1-phosphate, and glycerophosphocholine are associated with the coagulation conditions of milk in Swedish Red dairy cows [101]. In addition, metabolites, such as uracil and lactic acid, are thought to be able to predict milk traits, making it possible to analyze the milk traits from a metabolic perspective and discuss the potential functions for some of the detected networks [107]. These studies have established the association between metabolite biomarkers and milk or lactation stages (summarized in Table 5).

#### 5.2.2. Biomarkers for Milk Quality

Sundekilde et al. [102] found that acetate and novel metabolites, such as hippurate, isoleucine, butyrate, fumarate and β-hydroxybutyrate, are associated with milk quality by an NMR metabolomics approach. They further found that other metabolites are associated with SCC in milk samples from two Danish dairy breeds. High abundance of lactate, butyrate, isoleucine, acetate, and β-hydroxybutyrate and low abundance of hippurate and fumarate in milk are coupled with high levels of somatic cells, thereby providing some potential biomarkers (i.e., acetate) for milk quality under high SCC conditions [102]. Moreover, metabolomics sheds new light on highlighting interspecies differences from analyzing the metabolites. To find the metabolites uniquely shared by the milk of a horse, Jersey cow, camel, yak, goat, caprine, buffalo and dairy cow, studies were carried out using NMR, LC-MS, or GC-MS methods [108,109]. These studies have indicated the following: (1) glycine and valine are only present in the milk of goats, malic acid and talose are only present in the milk of cows, and hydroxyglutaric acid is only present in pasteurized milk [108]; (2) both fructose and glucose exist in ultra-high temperature-treated milk or cow milk [108]; and (3) succinic acid and choline can be indicators to differentiate cow milk from the milk from a horse, Jersey cow, camel, yak, goat, caprine, or buffalo [109]. These results validated that metabolomics is a feasible approach for milk typology analysis to reduce food fraud [108,110]. Therefore, these metabolites, generated in response to various conditions in milk, are good biomarkers in the evaluation of milk quality and safety (summarized in Table 5).

Although metabolomics is in its first stages in lactation research, combined with multivariate data analysis tools, it will help progress the study of lactation in the future due to its high-throughput abilities and high accuracy. The discovery of the unique metabolites of lactation as well as the biomarkers (the special metabolites) by these technologies would provide a better perspective for lactation research.

## 6. Conclusions and Future Directions

This review summarized the status of genomics, transcriptomics, proteomics, and metabolomics in lactation research mainly in dairy cows. When applying omics to lactation research, we must keep in mind that their advantages and limitations still depend on the cost, reproducibility and throughput. Thus, it is important to take your study aim and plans into consideration when you choose among these methods. Furthermore, these novel technologies combined with bioinformatics constitute a powerful tool to study the systems biology, which can generate large datasets for lactation sciences. Integrating omics studies will also greatly facilitate the discovery of key genes, proteins, and metabolites that function to regulate the metabolic pathways and the mechanisms of nutritional management on milk composition as well as facilitate the identification of biomarkers for mastitis. For instance, integrating omics could help us to simultaneously obtain enormous data (such as key genes, proteins, and metabolites) from mammary gland, blood or milk in dairy cows with high production and low production, various diets, etc. Thereafter, the function of identified key gene (coding the protein) can be further investigated in bovine mammary cells to reveal the mechanism of lactation and the novel biomarkers affected by some factors (such as physiology, nutrition, management, etc.) in dairy cows. Thus, while some technical or economic challenges remain, integrating omics still holds great promise in helping to enhance lactation performance.

## Figures and Tables

**Table 1:** The application of genomics in lactation research in dairy cows.

Methodology	Sample Source	Candidate Genes/Biomarker	References
Milk production			
A combination of mapping and molecular approaches	Cattle whole genome shotgun (WGS) downloaded from National Center for Biotechnology Information	The ras-related protein rap-1A on bovine chromosome 3, insulin-like growth factor 2	[12,13]
Milk composition			
Bayesian stochastic search variable selection model	Milk	Diacylglycerol-acyl transferase 1 (DGAT1), DGAT1 K232A, stearoyl CoA desaturase 1 A293 V polymorphisms, sterol response element-binding protein-1	[14,15,16]
Animal model in ASReml or capillary zone electrophoresis	Milk	β-casein (CN) genotype and the κ-CN genotype, variants of the β-lactoglobulin (LG) genotype B and the β-κ-CN haplotype A2B	[17,18]
Mastitis			
Genome-wide association studies	Blood	SNPs on Bos Taurus autosome 4 (BTA4), BTA18, and BTA6	[19]

**Table 2:** Summary of RNA sources used in the lactating bovine mammary gland transcriptome.

RNA Source	Composition	References
Mammary gland tissue	Mammary epithelial cells, myoepithelial, stromal and immune cells	[49,50]
Milk somatic cells	Lymphocytes, neutrophils, macrophages and exfoliated epithelial cells	[45,51]
Laser microdissected mammary epithelial cells	Selectively isolated epithelial cells from frozen tissue sections of the mammary gland	[48]
Milk fat globules	Cytoplasm of the mammary epithelial cells	[47]
Antibody-captured milk mammary epithelial cells	Exfoliated mammary epithelial cells	[41,47]

**Table 3:** Adaptations of expression profiles correlated to lactation stages.

Lactation Period	Features of the Mammary Gland	Transcriptomic Characteristics	References
Pregnancy	The morphogenesis of mammary ducts during early pregnancy and differentiation of the mammary alveolus during late pregnancy	Genes associated with cell cycle, cell proliferation, and the immune response	[55,56,57]
Initiation of lactation	Mammary differentiation, and proliferation, progressive expression of milk protein, and the secretion of precolostrum. The closure of the tight junctions between alveolar cells and the formation and secretion of colostrum and milk	Up-regulation of genes involved in milk synthesis concomitant with the inhibition of those related to cell proliferation. Some immune- and development-related miRNA highly expressed in colostrum and mammary glands	[40,55,58,59]
Middle lactation	Maintaining the number and activity of milk secreting cells	Milk constituents and milk synthesis-related pathways that are persistently expressed	[5,7,51,52,60]
Involution	The cessation of secretory activity and the reabsorption of milk residue, followed by a relatively static period. Invasion of leukocytes, increased epithelial cell death (through apoptosis or autophagy), and/or proliferation of connective tissue	A strong up-regulation of immune and antioxidant-related genes, and down-regulation of milk synthesis-related gene expression	[46,61]

**Table 4:** The application of proteomics in lactation research in dairy cows.

Methodology	Protein Type	Factors	Candidate Genes/Biomarker/Signaling	References
Milk components				
SDS-PAGE and MS	Milk fat globule membrane (MFGM)	Mid-lactation	Cell signaling and membrane/protein trafficking	[76]
Shotgun proteomics	MFGM	Day 7 after calving compared with colostrum	Lipid transportation, synthesis and secretion	[77]
2D and MS	whey proteome	The period of early lactation	Immunoglobulins and caseins	[78]
2D and MS	The main whey protein	Caprine, bovine, equine and buffalo	β-lactoglobumin	[79]
2D and MS	Milk protein	Colostrum	β2-microglobulin, Vitamin D-binding protein, zinc-α-2-glycoprotein and immunoglobulin G2 chain C	[78,80]
iTRAQ	Specific proteins	Various species	Clusterin (buffalo), biglycan (goat), quinone oxidoreductase and whey acidic protein (camel), clusterin (buffalo), primary amine oxidase (cow), uncharacterized protein (yak), high abundance of antimicrobial proteins (bovine), and high concentrations of the mucosal defense system (human)	[81,82]
Nutrition and lactation stages				
2D and MS	Milk protein	Lys or Met	A series of biological processes such as transcription, translation, protein synthesis, cell division and differentiation and even the cell cycle	[83,84]
2D and MS	Mammary gland protein	The lactation and non-lactation stages	Up-regulated proteins are involved in biological processes such as transportation, metabolism, biosynthesis, protein processing, the pentose-phosphate shunt, secretion and cell apoptosis; downregulated proteins play roles in other processes such as lipid degradation, transportation and the cytoskeleton	[85]
iTRAQ	Liver protein	Early lactation, and during mid-lactation	Isocitrate dehydrogenase and pyruvate carboxylase	[86]
iTRAQ	Liver protein	Physiological imbalance	Methylmalonate-semialdehyde dehydrogenase and alcohol dehydrogenase-4	[86]
FASP-Dimethyl Labeling-NanoLC- MS/MS	Milk protein	Energy balance	Galactose-1-phosphate and stomatin	[73]
Mastitis				
LC-MS/MS	Milk protein	Inflammation	Low-abundance proteins such as lactoferrin, transferrin, apolipoprotein AI, and fibrinogen	[74]
iTRAQ	Milk whey proteins	Infection with *Staphylococcus aureus*	Casein peptides, osteopontin, serum proteins, minor acute phase proteins and complement components	[87]
2D and MS or iTRAQ	N-linked glycosylated proteins	Clinical mastitis	High-abundance proteins such as hemoglobin β, cytochrome C oxidase, annexin V and α-1-acid glycoprotein as well as collagen type I α 1 and inter-α (Globulin) inhibitor H4	[23,88,89]

Abbreviations: SDS-PAGE, Sodium dodecylsulfate -polyacrylamide gel electrophoresis; 2-DE, two-dimensional electrophoresis; MS, mass spectrometry; iTRAQ, isobaric tags for absolute and relative quantification; LC-MS, liquid chromatography-mass spectrometry; FASP, filteraided sample preparation.

**Table 5:** The applications of metabolomics in lactation research in dairy cows.

Methodology	Candidate Genes/Biomarker	Unique Characterization	References
Milk composition			
NMR or MS analysis	Phosphorylated saccharides, acetone and β-hydroxybutyrate	Early lactation	[103,105]
GC/MS and LC/MS/MS	Hippurate and ribose 5-phosphate	Organic whole milk	[106]
NMR spectroscopy	Choline, *N*-acetyl hexosamines, creatinine, glycerophosphocholine, glutamate, glucose 1-phosphate, galactose 1-phosphate and orotate	Total protein content	[101]
NMR spectroscopy; GC-MS	Lactate, acetate, glutamate, creatinine, choline, carnitine, galactose 1-phosphate, and glycerophosphocholine, uracil and lactic acid	The coagulation conditions of milk and milk traits	[101,107]
Milk quality			
NMR metabolomics approach	Acetate and hippurate, isoleucine, butyrate, fumarate and β-hydroxybutyrate	Milk quality under high SCC conditions	[102]
GC-MS analysis	Glycine and valine	Goat milk	[108]
GC-MS analysis	Malic acid and talose	Cow milk	[108]
GC-MS analysis	Hydroxyglutaric acid	Pasteurized milk	[108]
GC-MS analysis	Fructose and glucose	Ultra-high temperature-treated milk or cow milk	[108]
NMR spectroscopy analysis and LC-MS spectrometry analysis	Succinic acid and choline	Cow milk	[109]

Abbreviations: MS, mass spectrometry; GC-MS, gas chromatography-mass spectrometry; LC-MS, liquid chromatography-mass spectrometry; NMR, nuclear magnetic resonance.
